# An Update on Resources, Procedures and Healthcare Provision in Pain Units: A Survey of Spanish Practitioners

**DOI:** 10.3390/ijerph18020451

**Published:** 2021-01-08

**Authors:** Mar Polo-Santos, Sebastián Videla-Cés, Concha Pérez-Hernández, Víctor Mayoral-Rojals, Mª Victoria Ribera-Canudas, Antonio Sarría-Santamera

**Affiliations:** 1 Agencia de Evaluación de Tecnologías Sanitarias del Instituto de Salud Carlos III-Avenida Monforte de Lemos 5, 28029 Madrid, Spain; 2 Spanish Network of Health Services Research and Chronic Diseases (REDISSEC) (MPS, ASS), 28029 Madrid, Spain; gris.hesereg@gmail.com; 3 Societat Catalana de Dolor, Academia Ciències Mediques de Catalunya i de Balears, 08017 Barcelona, Spain; svidelaces@gmail.com (S.V.-C.); mvribera@gmail.com (M.V.R.-C.); 4 Unidad de Soporte a la Investigación Clínica, Departamento de Farmacología Clínica, Hospital Universitario de Bellvitge-IDIBELL, L’Hospitalet del Llobregat, 08907 Barcelona, Spain; 5 Pharmacology Unit, Department of Pathology and Experimental Therapeutics, Faculty of Medicine and Health Sciences, IDIBELL, University of Barcelona, L’Hospitalet de Llobregat, 08907 Barcelona, Spain; 6 Sociedad Española del Dolor, Paseo de la Castellana 241, 28046 Madrid, Spain; cphernandez@salud.madrid.org (C.P.-H.); victormayoral@mac.com (V.M.-R.); 7 Unidad del dolor Hospital Universitario de la Princesa, 28006 Madrid, Spain; 8 Unidad de dolor Hospital Universitario de Bellvitge-IDIBELL, 08907 Barcelona, Spain; 9 Departament of Medicine, Nazarbayev University School of Medicine, Kerey and Zhanibek Khans St, Nur-Sultan 010000, Kazakhstan

**Keywords:** pain unit, assessment, questionnaire, multidisciplinary care

## Abstract

Multidisciplinary pain treatment units are recommended to provide comprehensive diagnosis and treatment of chronic pain, a complex clinical syndrome and one of the leading causes of disability worldwide. The objective of this study was to provide updated results on the situation of pain treatment units in Spain and to determine compliance with recommendations proposed by de Spanish Ministry of Health (SMH). A cross-sectional, prospective, multicenter survey was performed, collecting data on resources, procedures and healthcare provision. Between March and May 2019, the Spanish Pain Society sent an invitation letter to 183 pain units with a link to the questionnaire. Sixty-nine units from 13 regions agreed to participate. According to the International Association for the Study of Pain criteria, only 12 units were classified as multidisciplinary pain centers. Most (95.7%) were in hospitals, 82.6% from the public sector, and 46.4% had protocols to coordinate with primary care. Interviewees rated the adequacy of facilities at 6.3 (from 0 to 10). Moreover, 67% of interviewees found that there were insufficient staff, with no mental health professionals, physical therapists or social workers in 49.3%, 87.0% and 97.1% units, respectively. Only 24 pain units had a day hospital, 44.9% offered psychological interventions, and 79.7% supported teaching and research activities. Results suggest that a small proportion of Spanish pain units meet the national standards for multidisciplinary pain units proposed by the SMH.

## 1. Introduction

Pain units have been proposed as critical components to provide the multidisciplinary and comprehensive care required to address chronic pain, a complex clinical syndrome, based on a biopsychosocial model of pain [1]. A biopsychosocial model is a conceptual model that proposes that psychological and social factors should also be included along with biological variables in the understanding of the medical illness; in fact, each patient has his or her own thoughts, feelings and history [2]. Hence, the biopsychosocial model for pain management should include physical (biological), psychological, social, cognitive, affective and behavioral measures—along with their interactions—to best assess the individual’s unique pain condition. The International Association for the Study of Pain (IASP) has developed recommendations for pain units, including that they should incorporate highly skilled clinicians from different disciplines (e.g., physicians, nurses, mental health professionals (i.e., psychologists or psychiatrists) and physical therapists), have appropriate facilities and guarantee that professionals work in the same space and communicate on a scheduled basis [1,3].

Chronic pain, defined as pain on most days or every day in the previous six months [1], is one of the leading causes of disability and disease burden worldwide [4]. Globally, it is estimated that approximately 20% of the population suffers from chronic pain, and, because older patients experience chronic pain more frequently, population ageing will increase this prevalence [4]. In Spain, the Official National Health Survey showed that 23.3% of the general population suffered moderate or severe pain in 2017, and this proportion reached up to 40% among older individuals [5]. Pain is associated with elevated direct but also indirect costs, because of the productivity loss in the working-age population [6].

Pain units have to adapt to the increasing complexity of pain. Isolated interventions (e.g., opioid medication or surgeries) have many times been shown to lack long-term benefits and even produce risks, as they obviate the need for a more holistic approach. More sophisticated therapies have been developed based on the understanding that pain is a multifaceted phenomenon in which physiological, psychological and social factors interact. There is strong evidence that multidisciplinary management units offer the best clinical care and are the most cost-effective long-term treatment options [7].

A periodic assessment of the organizational performance of pain treatment facilities, including the structure and functional aspects of pain units, constitutes a helpful tool to understand how these units adhere to the recommendations for multidisciplinary pain management. In Spain, two major previous surveys revealed a high dependence of pain units on anesthesiology services, a lack of dedicated resources and a part-time dedication of most practitioners serving at these units [8,9]. The main objective of this study was to provide updated results on the current situation of pain treatment units in Spain and to identify the level of compliance with the national recommendations proposed by the National Ministry of Health.

## 2. Materials and Methods 

### 2.1. Study Design

This was a cross-sectional, prospective, multicenter survey study, based on a questionnaire created ad hoc. 

The final study protocol was approved by the Clinical Research Ethics Committee of Bellvitge University Hospital in Barcelona (reference: PR032/19). The need for written informed consent was waived. This study was carried out according to the stipulations of the Declaration of Helsinki, and the level of protection of confidentiality concerning the protection of personal data as required by Spanish laws (LOPD 3/2018) was ensured. 

### 2.2. Questionnaire

Based on previous surveys [8,9,10] and on the national standards proposed by the Spanish Ministry of Health [7], the study coordinators developed the questionnaire with 35 items, which collected information on space, equipment, staff composition, treatments offered, caseload, waiting times and participation in teaching and research activities. 

Between March and May 2019, an invitation letter to participate was sent by the chairperson of the Spanish Pain Society to a census of 183 chronic pain units together with a link to the questionnaire. Web links were personalized so that they could be completed only once per site. The details of the production of this census are available elsewhere [10]. 

### 2.3. Statistical Analysis

No formal sample size was calculated. The sample size was defined as the total number of pain units which responded to the questionnaire. 

A descriptive analysis was performed. Medians (interquartile ranges (IQR)), percentages and 95% confidence interval (95%CI) were calculated for continuous and categorical variables, respectively. A map of the spatial distribution of pain units was drawn using the “spmap” package in Stata vs. 13.0 [11].

### 2.4. Compliance with Spanish Standards

A “traffic light system” was developed to assess the level of compliance of the responses received with the Spanish Standards and Recommendations criteria for functional, structural and organizational requirements for pain units [12], where green was assigned when ≥75% of pain units reported meeting that standard; yellow in the case of ≥50%–<75% compliance; and red in the case of <50%.

## 3. Results

**Figure 1:**
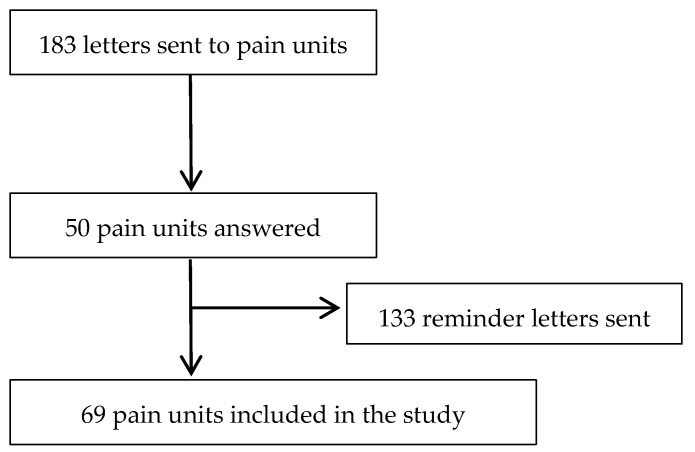
Study flowchart.

**Figure 2:**
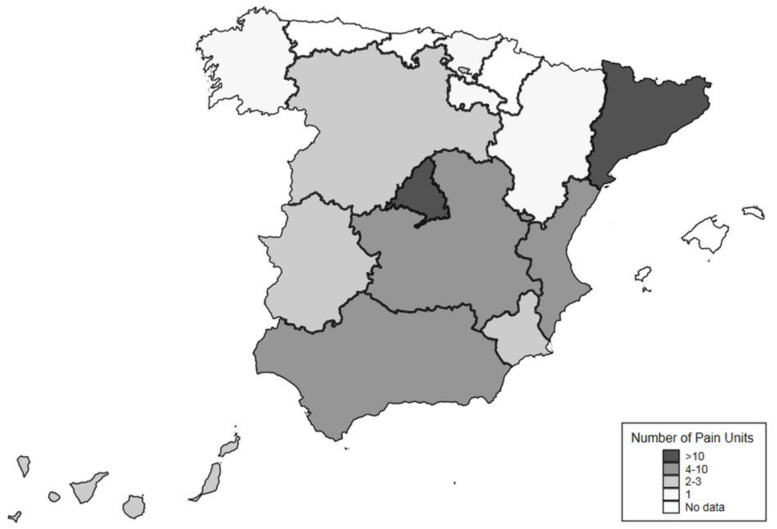
Number of surveyed pain units per autonomous community (region).

Sixty-nine pain units (Figure 1) from 13 autonomous communities (regions) agreed to participate and were included in the analyses (please see Figure 2). Following the IASP guidelines, 38 pain units were classified as modality-oriented clinics (i.e., healthcare facilities that offered a specific type of pain treatment but did not provide comprehensive assessment or management); 19 as pain clinics (i.e., facilities which offered pain treatment and comprehensive assessment and management, but were not multidisciplinary); and 12 as multidisciplinary pain centers (i.e., facilities with a variety of care providers capable of assessing and treating physical, psychosocial, medical, vocational and social aspects of chronic pain). Response rate was 38%. 

The most frequent survey respondents (84.1%) were the directors/coordinators/heads of the pain centers, which in most cases (92.8%) were anesthesiologists. Their median (IQR) time of clinical experience with patients with pain was 20 (14–25) years. Most units (95.7%) were integrated into hospitals, 82.6% were from the public sector, and 46.4% had established protocols to coordinate with primary care services establishing specific circuits to identify the cases with the highest priority (93.7%) while avoiding unnecessary referrals (90.6%). Twenty-six centers (37.7%) provide also acute pain services, and in 80.8% of cases was also managed by the pain unit. Fifty-five of the pain units surveyed (79.7%) were part of the Anesthesia and Reanimation units. Fourteen units (20.3%) had certified their management system according to ISO standards and 32 interviewees (46.4%) considered that their pain unit met the national standards as proposed by the Spanish Ministry of Health [7], while 29 units (42%) only met partially those standards.

As for their facilities, on average, interviewees rated the adequacy 6.3 on a 0 to 10 scale. The median (IQR) size of pain treatment facilities was 100 m^2^ (6–400 m^2^). Information on the attributes of pain treatment units is provided in Table 1. All units had at least one dedicated outpatient consulting room (median number of two rooms per unit), 89.9% had a reception room, 72.5% had at least one operating theater (available for a median number of two interventions in the mornings and one in the afternoons), 71.0% had access to block rooms, 66.7% had access to hospital beds in the event of complications, 50.7% had an administrative area, and 50.7% had a nursing station. Only 24 pain units (34.8%) had their own day hospital. Facilities for monitoring, respiratory support and resuscitation were available in 89.9% and 84.1% of the pain units, respectively. Although most units had adequate Information Technology systems, only 49.3% had specific computerized medical records for chronic pain management. All operating rooms were equipped with patient monitoring systems, appropriate instrumentation for nerve blocks, X-ray image intensifiers and radiology personal protective equipment. 

**Figure 3:**
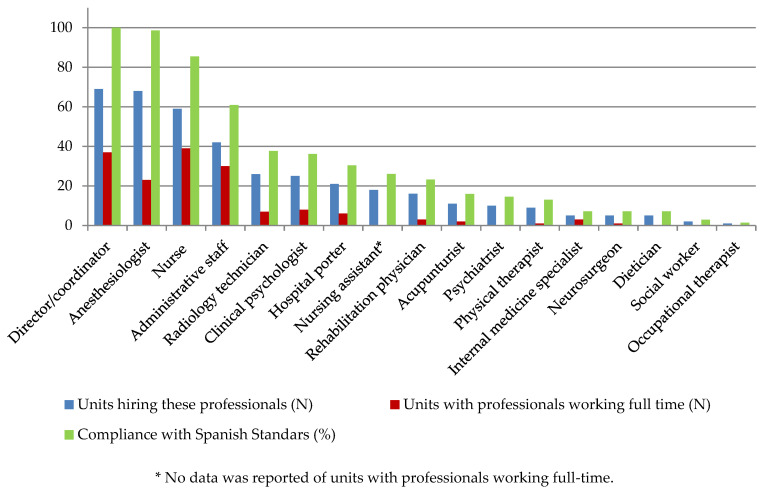
Availability of professionals in a sample of Spanish pain units in 2018 (*n* = 69).

Staff composition can be found in Figure 3. There was a median (IQR) of 10 (7–14) professionals working at each unit, six (4–9) of whom were not full-time employees. Along with anesthesiologists (present in 98.6% of the pain units), nurses (85.5%) and administrative staff (60.9%) were the most common professionals working in the units. This table shows also the level of compliance of those criteria with the Spanish Standards.

Sixty-seven percent of interviewees felt that the staff was insufficient and most pain units lacked mental health professionals (clinical psychologist or psychiatrists) (49.3%), physical therapists (87.0%), social workers (97.1%) or occupational therapists (98.6%). 

During 2018, the median (IQR) number of first-time visits in the studied units was 700 (400–1334) and of follow-up was 1987.5 (940.5–3724) (please see Table 2). Most units received patients from inside (95.7%) and outside (82.6%) the catchment area of their hospital. The professionals that most frequently referred patients for their first appointment to the units were traumatologists (38.7%), general practitioners (12.8%), neurosurgeons (11.8%) and rehabilitators (8.6%). The median (IQR) number of diagnostic techniques was 216 (15–650). Regarding treatments, all units offered pharmacotherapy and interventional therapies. The latter included, but were not limited to, intra-articular and peripheral nerve blocks (100% of units offered this service), head and neck blocks (98.6%), central blocks (97.1%), trunk nerve blocks (97.1%), radiofrequency (91.3%), sympathetic nerve blocks (87.0%), subarachnoid blocks (75.4%), neurolysis (68.1%), implant technologies (62.3%), ablative neurosurgical procedures (26.1%) and epiduroscopy (24.6%). Less than half of the units (44.9%) offered psychological treatment. Among units who had performed outpatient, surgical or radiological interventions in 2018, these ranged from 24 to 1041, 74.5 to 750 and 0 to 300, respectively (Table 3). Although these varied according to patient severity, the median consultation times for a standard first appointment or a follow-up visit were 30 (30–45) and 15 (15–20) min, respectively; and the median consultation time for an in-clinic interventional visit was 30 (22.5–45) min. At discharge, 63.8% and 76.8% of units provided patients with written recommendations or a direct telephone number in case of emergency, respectively. Only 23% units surveyed patients about their perceived quality of healthcare.

Table 3 presents information on education and research services provided by pain units. In 2018, 39 (56.5%) units had published at least one international article and 29 (42.0%) units had received international research funding within the previous 5 years. A total of 55 (79.7%) units supported teaching activities, of which more than two thirds received (66.7%) rotating physicians (mainly anesthesiology and primary care residents) and organized regular clinical sessions to discuss patient’s treatment options.

The overall level of compliance with the Spanish Standards and Recommendations criteria for functional, structural and organizational requirements for pain units as estimated by the “traffic light system” showed still some relevant room for improvement, with several areas in yellow and even in red.

## 4. Discussion

The present survey provides an updated description of the main characteristics of a sample of 69 pain units from 13 autonomous communities (regions) in Spain. The main finding is that a small proportion of Spanish pain units meet the national standards for multidisciplinary pain units proposed by the SMH. 

Our findings are in line with those of previous surveys [8,9] and reveal that recent efforts to improve pain management in the Spanish National System, as described in the 2011 National Standards and Recommendations for Quality and Safety in Pain Units [7] or the 2014 Framework Document for the improvement of pain management in the Spanish National Health System [12], have not resulted in sufficient improvements in pain units. One example of how these national recommendations have not been adequately implemented is seen in the requirement for pain units to have a designated space. 

Spanish Standards and Recommendations established criteria for functional, structural and organizational requirements that guarantee adequate safety, quality and efficiency conditions to care for patients with chronic pain who require specialized assistance.

Pain units must have appropriate human resources, including a director, a professional with advanced training in the study and treatment of patients with chronic pain, a care coordinator, doctors with training in pain management and support staff, which includes nurses, pharmacists, physical therapists, psychologists, occupational therapists and social workers with specialized training in pain.

The unit should have an organization and operation manual. It is recommended that the unit has a patient registry for continuity of care. The Spanish guidelines also establish recommendations for the availability of a treatment room and block and operating rooms for the appropriate management of patients depending on the size of the center and the volume of patients [7].

Our results suggest that most still share their space with other hospital or healthcare services. Another instance of the recommendations not being fulfilled is that although they highlight the importance of pain units being led by a multidisciplinary team composed of different healthcare professionals including doctors, nurses, psychologists and physiotherapists, and of this team being directed by a full-time physician with extensive experience in pain management, these requirements have not been met [7]. Our results on this matter show that most teams are led by part-time physicians, mainly composed of doctors and nurses, with a lack of other professionals such as clinical psychologists, physical therapists, social workers or occupational therapists.

Regarding waiting times, we observed that these were longer than the recommended national and international guidelines. Thus, for example, the IASP establishes maximum waiting times of 1 week for most urgent conditions including cancer-related pain, 1 month for urgent or semi-urgent pain with risk of increasing functional impairment and 2 months for non-urgent appointments [13]. 

Another aspect that deserves attention is the relatively low proportion of units that have implemented specific computerized medical records for chronic pain management, which are essential for assessing individual treatment outcomes and evaluating overall program effectiveness. Moreover, it is important to note that only 50% of units had protocols in place on coordinating with primary healthcare, despite national standards urging units to establish strong coordination with general practitioners. Promoting continuity of care between specialized pain units and primary care improves quality of care, patient satisfaction and decreases further hospitalizations, which could have a significant impact on improving patients’ outcomes [14]. 

An unexpected and positive finding was the high proportion of pain units that performed teaching and researching activities, with more than half of them reporting having been granted research projects or having published at least one international article during the last 5 years. In addition, a high proportion of units (66.7%) reported receiving rotating physicians, most of them being anesthesiology medical residents. 

These findings are, unfortunately, not an exception but common, at least in the European landscape. In Ireland, the number and structure of pain units is still not optimal. A national strategy on acute pain to coordinate and focus resources in providing acute pain management is proposed [15]. The availability of pain units in Germany has increased over the last decade; however, the quality of nearly half of those units is questionable [16]. A similar finding was noted in the United Kingdom, where many pain services do not meet minimal national standards [17]. Significant variability has been identified in the Netherlands, both in the way in which pain units are organized and in the activities which they employ [18]. Moreover, Portugal has not yet achieved the goal outlined by recent governmental guidelines that mandates the existence of a pain unit with a structured organizations model in every hospital with surgical activity [19]. 

Although different countries and healthcare systems may have variations in their own quality criteria for pain units, the present results in Spain confirm what has been found in the international literature and demonstrate the lack of a commonly accepted definition of what constitutes a paint treatment unit. The focus should now be on human resources, education and training of the personnel and the other disciplines involved, as well as a multidisciplinary, multi-professional approach to delivering high-quality services. Continued investment in these services is key to supporting patients with complex pain and may potentially reduce the high prevalence of persistent pain and its effects on lower quality of life and elevated use of resources, representing not just a significant direct but also an indirect burden on our societies [6].

The main limitation of the present study is its low response rate (38%), which may limit its national representativeness and may induce selection bias if there are differences in size and resource availability between the pain units which responded to the questionnaire and those which did not. It seems that larger hospitals are more represented among those which responded to the questionnaire, and therefore, the results of the study could overestimate the overall availability of resources, which would have an impact on the percentage of compliance with national recommendations as it would be even lower. Another limitation refers to the data collection methodology through self-administered questionnaires, which increases the risk of information bias. However, these surveys have shown to be a helpful tool in understanding the adherence of pain units to the recommendations for multidisciplinary management. Moreover, by using a similar methodology, we could compare our results with those of previous surveys. 

## 5. Conclusions

In summary, our results suggest that only a small proportion of Spanish pain units meet the national recommendations proposed by the National Ministry of Health. Due to the extensive evidence that multidisciplinary care is the most cost-effective intervention for chronic pain, more efforts are needed to achieve these standards. 

## Figures and Tables

**Table 1:** Facilities and resources available in a sample of Spanish pain units in 2018.

	Units *n* (%) 95%CI	Compliance with Spanish Standards
**FACILITIES (*n* = 69)**		
**Which facilities are available at the pain clinic?**		
Consultation/treatment rooms for ambulatory visits	69 (100)	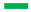
Waiting room	62 (89.9) (0.83–0.97)	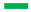
Operating theater	50 (72.5) (0.62–0.83)	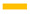
Access to block rooms	49 (71.0) (0.60–0.82)	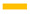
Independent management of inpatient beds	46 (66.7) (0.56–0.78)	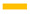
Room for clinic sessions/meetings/library	36 (52.2) (0.40–0.64)	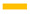
Staff area (office, wardrobe, etc.)	36 (52.2) (0.40–0.64)	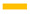
Administrative area	35 (50.7) (0.39–0.63)	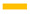
Nursing station	35 (50.7) (0.39–0.63)	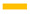
Own day hospital	24 (34.8) (0.24–0.46)	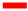
**EQUIPMENT AND SERVICES**		
**Which material resources are available in the pain unit? (*n* = 69)**		
Patient basic monitoring equipment	62 (89.9) (0.83–0.97)	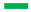
Life support and emergency resuscitative equipment	58 (84.1) (0.75–0.93)	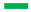
Internet access	67 (97.1) (0.96–0.99)	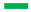
Access to electronic records	65 (94.2) (0.02–0.15)	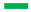
Integrated information systems to register their activity	64 (92.8) (0.87–0.99)	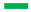
Direct telephonic access	61 (88.4) (0.81–0.96)	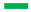
Access to physical and online resources from library databases	55 (79.7) (0.70–0.89)	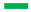
Computerized records for chronic pain management with exclusive access for the pain unit	34 (49.3) (0.38–0.61)	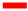
**Which material resources are available in the operating theater of the pain unit? (*n* = 50)**		
Patient monitoring equipment	50 (100)	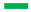
Instrumentation for nerve blocks	50 (100)	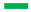
X-ray image intensifier	50 (100)	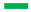
Radiology personal protective equipment	50 (100)	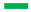
Local imaging facilities (ultrasound)	48 (96.0) (0.38–0.61)	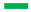
Anesthesia equipment: respirator	46 (92.0) (0.01–0.16)	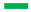
Radiofrequency devices	45 (90.0) (0.82–0.98)	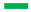

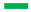
 ≥75%; 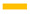 ≥50%–<75%; 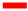 <50% of compliance with Spanish Standards.

**Table 2:** Healthcare provision in a sample of Spanish pain units in 2018 (*n* = 69).

	Median (IQR)	*n* (%) (95%CI)	Compliance with Spanish Standards
**HEALTHCARE SERVICES PROVIDED**			
Number of first-time appointments	700 (400–1334)		
Number of follow-up appointments	1987.5 (940.5–3724)		
Number of diagnostic techniques applied	216 (15–650)		
Number of outpatient intervention techniques applied	300 (24–1041)		
Number of surgical intervention techniques applied	426 (74.5–750)		
Number of radiological intervention techniques applied	0 (0–300)		
**CONSULTATION AND WAITING TIMES**			
Consultation time for standard first appointment (minutes)	30 (30–45)		
Consultation time for standard follow-up appointment (minutes)	15 (15–20)		
Consultation time for non-interventional pain treatment (minutes)	20 (15–30)		
Consultation time for interventional pain treatment (minutes) ^a^	30 (22.5–45)		
Actual maximum waiting time for first patient appointment:			
Oncologic patients (days) ^b^	7 (4–15)		
Urgent appointments (days) ^c^	15 (7–30)		
Non-urgent appointments (days) ^d^	60 (30–180)		
Actual maximum waiting time for successive patient appointment:			
Oncologic patients (days) ^b^	15 (7–30)		
Urgent appointments (days) ^c^	25 (7–40)		
Non-urgent appointments (days) ^d^	90 (21–180)		
**SHARING OF INFORMATION WITH PATIENTS**			
The patients receive written recommendations about their pathology		44 (63.8) (0.52–0.75)	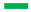
The patients are provided with a direct telephone number to ask questions		53 (76.8) (0.67–0.87)	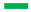
A written informed consent is requested for all interventions performed		69 (100)	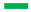
The patients are surveyed about the perceived quality of healthcare		16 (23.2) (0.13–0.33)	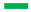

^a^ Only 48 pain units offered these services. ^b^ Only 59 pain units treated oncologic patients. ^c^ 66 pain units attended urgent appointments. ^d^ 67 pain units attended non-urgent appointments. 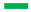 ≥75%; of compliance with Spanish Standards.

**Table 3:** Medical education and research activities in a sample of Spanish pain units (*n* = 69).

	Units *n* (%) (95%CI)	Median (IQR)	Compliance with Spanish Standards
Presentations to congresses within the last 5 years (2014–2018)			
National	60 (87.0) (0.79–0.95)	4 (3–6)	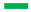
International	44 (63.8) (0.52–0.75)	2 (1–3)	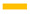
Publications within the last 5 years (2014–2018)			
National	47 (68.1) (0.57–0.79)	2 (1–2)	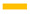
International	39 (56.5) (0.45–0.68)	1 (1–6)	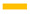
Research projects within the last 5 years (2014–2018)			
National	48 (69.6) (0.59–0.80)	1.5 (1–2)	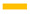
International	29 (42.0) (0.30–0.54)	1 (1–2)	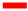
Rotating physicians during the last year (2018)	46 (66.7) (0.56–0.78)		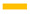
Resident physicians	N.R.	4 (2–8)	
Non-resident physicians	N.R.	2 (0–6)	
Training activities during the last year (2018)	55 (79.7) (0.70–0.89)	N.R.	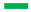
Hospital general sessions (*n* = 55)	45 (81.8) (0.72–0.92)	N.R.	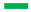
Courses to physicians from other departments of the hospital (*n* = 55)	50 (90.9) (0.83–0.99)	N.R.	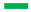
Courses to primary care physicians (*n* = 55)	48 (87.3) (0.79–0.96)	N.R.	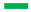

N.R: not reported. 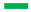 ≥75%; 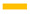 ≥50%–<75%; 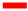 <50% of compliance with Spanish Standards.

## Data Availability

The data that support the findings of this study are available from the corresponding author upon reasonable request.
